# Invited Papers 2

**DOI:** 10.1038/sj.bjc.6601925

**Published:** 2004-06-22


**British Journal of Cancer (2004) 91**(Suppl 1), S3 – S7
© 2004 Cancer Research UK All rights reserved 0007 – 0920/04 $30.00
**www.bjcancer.com**

### INVITED PAPERS 2

### S1
### COMPLEX BIOLOGICAL RESPONSES TO DAMAGING AGENTS
Leona D. Samson, Biological Engineering Division,
Center for Environmental Health Sciences, Massachusetts Institute of Technology, USA

Alkylating agents are abundant in our cellular and external environment, and they are toxic, mutagenic, and carcinogenic. Because of their toxic properties, certain alkylating agents are also used for cancer chemotherapy. Therefore, it is important to elucidate the constellation of different biochemical and genetic pathways by which both normal and malignant cells respond to alkylation damage. Our ultimate goal is to develop ways of predicting how certain agents will affect these very different target cell populations. It is well known that a variety of DNA repair pathways and cell cycle checkpoint pathways protect cells against the toxic effects of alkylation damage. But now we have shown that a multitude of other unexpected pathways are also involved in cellular recovery, and our goal is to understand how these cellular pathways operate and interact with each other to determine the ultimate phenotypic response to such environmental assaults. We use a variety of cellular and animal model systems ranging from bacteria and yeast to mice and humans. We have studied global genomic responses to alkylation damage in *E. coli* and *S. cerevisiae*, in mouse and human cell lines, and in various tissues of exposed mice. The results demonstrate that global cellular responses to alkylation damage are far more complex than first thought and some of the unexpected pathways that protect against alkylation-induced toxicity will be presented and discussed.

### S2
### CANCER GENE THERAPY: GETTING VECTORS, GENES AND T CELLS TO TUMOURS.
Gregory Daniels, Luis Sanchez-Perez, Timothy Kottke, Rosa Maria Diaz, Caroline Cole, Michael Forshaw, Jill Thompson, Michael Gough, Jian Qiao, Richard G. Vile, Molecular Medicine Program and Department of Immunology, Mayo Clinic, Rochester, Minnesota 55905. This lecture has been kindly sponsored by NTRAC.

Our long term interest is in the development of truly systemic gene delivery systems which do not require prior knowledge of the location of sites of metastatic disease to address the most challenging aspects of gene therapy of cancer, where accessibility of a tumor is not a pre-requisite of successful gene delivery/therapy.
We have pursued two separate routes to achieve this goal. In the first, we have devised protocols in which vectors can be carried to local sites of tumor growth and be released in response to externally applied signals (such as drugs) or environmental cues supplied by the biological properties of the tumor itself. We have developed the concept of T cell carriers for the gene therapy of cancer using the B16ova murine model in which B16 cells stably express the ovalbumin model antigen. Adoptive transfer of OT-1 T cells, transgenic for the T cell receptor that recognizes the SIINFEKL epitope of ova in the context of presentation by H2Db+ve B16ova cells leads to tumor killing *in vivo*. To convert the OT-1 T cells into carriers of retroviral vectors we have exploited an observation that retroviral particles will adhere to the external cell surface without necessarily productively infecting the target cell. OT-1 T cells loaded with surface coated retroviral stocks hand the virus off to new target cells in culture dependent upon the presence of a functional envelope whose receptor is only poorly, or not, expressed on the T cell itself. In addition, adoptive transfer of OT-1 T cells loaded with surface coated retrovirus encoding an HSVtk gene led to significant improvements in survival of mice bearing lung metastases of B16ova tumor cells compared to OT-1 cells alone. Therefore, it is possible to enhance the therapeutic potential of adoptive T cell transfer by combining tumor localized production of cytotoxic retrovirus with the T cells' natural effector functions.
In the second approach we hypothesised that *in situ*, inflammatory killing of a normal tissue from which a tumor derives may generate autoimmune reactivity to self antigens expressed in that tissue as well as in the tumor cells. We have now shown that simple intradermal injection of 2 plasmids promoted tissue specific, inflammatory killing of melanocytes and induced an immune response that eradicated systemically established B16 tumors. The therapeutic response was rapidly suppressed *in vivo* and did not necessarily induce autoimmune disease. This is the first time that deliberate destruction of normal tissue has been used to generate an effective immune response against malignant disease.
In summary, our results show that it will be possible to deliver genes to normal, or tumor, cells such that significant anti tumor therapeutic effects can be achieved even in clinical situations where the location of the disease is unknown.

### S3
### REPLICATING ADENOVIRUSES THAT TARGET COLON CANCER
Christophe Fuerer, Krisztian Homicsko, Alex Lukashev and Richard Iggo.
Oncogene Group, Swiss Institute for Experimental Cancer Research (ISREC), 1066 Epalinges, Switzerland.

The Wnt pathway is constitutively activated in colon tumours by mutations in the adenomatous polyposis coli and β-catenin genes. We have developed adenoviruses that selectively replicate in cells with activated Wnt signalling by inserting binding sites for the Tcf family of transcription factors in the early promoters of human adenovirus 5. When Tcf sites are inserted in the E1A, E1B and E4 promoters, the virus shows 100 to 10,000-fold selectivity for cells with activated wnt signalling in viral burst and cytopathic effect assays. Despite evidence for efficacy in vitro, clinical studies with other oncolytic adenoviruses have shown that they are safe but lack efficacy. To selectively increase the toxicity of Tcf-regulated viruses we have expressed the yeast cytosine deaminase gene (yCD) after the fibre gene in the major late transcript using an internal ribosome entry site (IRES). Cytopathic effect assays in colon cancer cell lines show that the yCD virus has ~10-fold increased toxicity in the presence of the prodrug 5-fluorocytosine (5-FC), which is converted to 5-fluorouracil (5-FU) by yCD. Toxicity is higher following addition of 5-FC immediately after infection and viral burst size is only slightly reduced in the presence of 5-FC, showing that expression of yCD as a late gene is compatible with virus replication. Systemic therapy with $10^{11}$ particles of a Tcf-regulated virus leads to a significant delay in growth of subcutaneous SW620 xenografts, but FISH for viral DNA in treated tumours shows that virus spreads poorly within the tumour. To achieve cures with oncolytic adenoviruses will require concerted approaches to improve virus delivery to the tumour, spread within the tumour and killing of tumour cells.

### S4
### ONCOVEX: A FAMILY OF ONCOLYTIC HERPES SIMPLEX VIRUSES OPTIMISED FOR THERAPEUTIC USE
R Coffin, BioVex Ltd, Oxford, OX14 4RX

HSV in which ICP34.5 is deleted gives tumour selective replication *in vitro* and *in vivo*. Such viruses have also proven safe in Phase I clinical trials by intra-tumoral injection. However, oncolytic HSV containing an active gene has not previously been tested in man. Previous work has used serially passaged laboratory isolates of HSV which are attenuated in tumour cells as compared to clinical isolates. To enhance anti-tumour properties, we have deleted ICP34.5 from clinical isolates of HSV-1 giving greatly enhanced tumour cell killing. One of these viruses was then deleted for ICP47 (which blocks antigen presentation) and GM-CSF inserted to maximize anti-tumour immune responses following intra-tumoural injection. As expected, the anti-tumour properties of this virus were greatly improved by each of the modifications. *In vivo*, both injected and non-injected tumours could be cured and animals are protected against further tumour challenge. Following this data, a Phase I clinical trial with the virus (OncoVEX$^{GM\text{-}CSF}$) is underway including patients with cutaneous or sub-cutaneous deposits of a number of tumour types giving promising results so far. In addition, further versions of the virus expressing other genes have been constructed. These include viruses expressing TNF∝ and TNF∝ combined with GM-CSF, intended to be synergistic with radiotherapy, and versions expressing pro-drug activators and/or a fusogenic glycoprotein to maximize local tumour control. Each of these have shown promise in pre-clinical models, including in combination with chemotherapy. In summary, therefore, continued development of oncolytic HSV has provided a family of viruses incorporating a range of active genes intended to maximize the effects of the virus under the different circumstances for which oncolytic virus therapy might be considered.

### S5

### HYPOXIA MEDIATED ALTERATIONS IN THE THRESHOLD FOR DRUG-INDUCED APOPTOSIS TUMOURS

Caroline Dive, Janine T Erler and Ian J Stratford, Cellular and Molecular Pharmacology Group, Paterson Institute for Cancer Research, and School of Pharmacy, University of Manchester.

Drug resistance, an obstacle to curative treatment of solid tumours can occur via suppression of apoptosis, a process controlled by pro- and anti-apoptotic members of the Bcl-2 family. These proteins integrate a variety of cellular survival and stress signals including perturbations in oxygen supply and drug-induced damage. Solid tumours with disorganised, insufficient blood supply contain drug-resistant hypoxic cell subpopulations. We sought to determine the impact of oxygen deprivation on Bcl-2 family members and drug response in human colon cancer cells. Oxygen deprivation *in vitro* reduced mRNA and protein levels of pro-apoptotic Bid and Bad but had no effect on Bak. Hypoxia inducible factor-1 (HIF-1) was dispensable for the down regulation of Bad but it was required for Bid down regulation. This was consistent with HIF-1$\alpha$ binding to a hypoxia responsive element (-8484 to -8475) in the *bid* promoter. Oxygen deprivation resulted in proteosome-independent Bax down regulation *in vitro* and a global reduction of translation. The physiological relevance of Bid and Bax down-regulation was confirmed in a xenograft model. Oxygen deprivation resulted in reduced drug-induced apoptosis and clonogenic resistance to chemotherapeutic drugs with disparate mechanisms of action. The functional relevance of Bid and/or Bax down-regulation to drug response was evidenced by the relative resistance of normoxic cells that had no or reduced expression of Bid and/or Bax, and that forced expression of Bid in hypoxic cells resulted in increased sensitivity to etoposide. These data add impetus to the design of chemotherapeutic strategies to target HIF-1. Experiments to evaluate the impact of small molecule inhibitors of HIF-1 function on Bcl-2 family regulation of apoptosis in solid tumours are eagerly anticipated.

### S7

### THE PROMISE AND CHALLENGE OF TARGETING BCL-2 ANTI-APOPTOTIC PROTEINS IN CANCER

Jason O'Neill, Michael Manion, Pam Schwartz, David M. Hockenbery.
Fred Hutchinson Cancer Research Center, Seattle, USA

Cancer cells with elevated levels of BCL-2 and related survival proteins are broadly resistant to cytotoxic agents. Antisense oligodeoxynucleotides, and more recently small molecule ligands for BCL-2 and BCL-$X_L$ are directly cytotoxic or synergistic with standard cytotoxic agents, and in some cases, appear to have substantial selectivity for tumor cells. The usual issues for rational drug discovery are writ large upon BCL-2-targeted therapeutics. The molecular functions of BCL-2 are not well understood, such that validation of cytotoxicity related to BCL-2 as well as identification of surrogate markers for BCL-2 function, are significant obstacles for drug development. In these circumstances, small molecule inhibitors can help to critically evaluate current models for BCL-2 survival functions. An accepted premise for protein function in the BCL-2 family is the regulated association of survival and pro-apoptotic members as dimers and oligomers, employing BH3 domains and BH3-binding pockets on opposing subunits. Instead, the preservation of two binding sites for a BH3-binding pocket ligand, 2-methoxy antimycin A, in homodimers of BCL-$X_L$, suggested an alternative dimerization mechanism. An x-ray crystal structure of BCL-$X_L$ homodimers reveals a unexpected 3-D domain swapping mechanism with exclusion of the BH3-binding pockets from the dimer interface. A second tenet in mammalian apoptotic pathways is the dependence of BCL-2 function on the pro-apoptotic BAX and BAK proteins. Though this may be the usual relationship, studies with inhibitors show it is not without exception.

### S6

### DETERMINING THE REQUIREMENTS FOR TUMOR MAINTENANCE *IN VIVO*

Gerard I. Evan, Lamorna Brown Swigart, Maria Christophorou, Laura Soucek, Beth Lawlor, Josina Reddy, Daniel Murphy
UCSF Cancer Center, 2340 Sutter St., San Francisco, California 94143-0875.

Activation of the c-Myc oncoprotein is an archetypical proliferation-deregulating neoplastic lesion present in most human tumors. However, c-Myc also has potent apoptotic activity which intimates that c-Myc can only drive tumorigenesis in cells in which apoptosis is suppressed. To determine the minimal requirements for c-Myc-induced neoplasia *in vivo* we developed mice in which a reversibly switchable c-Myc oncoprotein has been targeted to specific tissues. This allows us to explore both the immediate and the delayed consequences of acute activation of c-Myc in different somatic settings *in vivo*, as well as assess the requirement for sustained Myc activation in tumor maintenance. When targeted to pancreatic cells, activation of c-Myc in the absence of any other oncogenic lesion induces 100% sustained proliferation of all cells in all islets. However, such cell proliferation is accompanied by overwhelming apoptosis that rapidly overcomes cell gain and expeditiously leads to islet involution and concomitant acute diabetes. This argues that c-Myc-induced cell neoplasia cannot occur without concomitant suppression of apoptosis. We confirmed this by co-expressing the anti-apoptotic Bcl-$x_L$ protein in cells, whereupon activation of c-Myc triggers rapid, progressive and inexorable cell neoplasia that was immediately accompanied by profound and progressive angiogenesis and invasion. Similarly, activation of c-Myc in cells lacking the tumor suppressor $p19^{ARF}$ or p53 triggers immediate tumorigenesis. Subsequent de-activation of the switchable c-Myc oncoprotein triggers rapid and complete regression of all cell tumors, together with their attending vasculature, indicating that Myc is required both to induce and to maintain the neoplastic state. Thus, activation of c-Myc is sufficient to confer multiple and diverse neoplastic traits that become overt only upon suppression of cell death. The immense complexity of the tumor phenotype appears to imply that numerous and diverse mutations are required for establishment and maintenance of cancers. However, our studies indicate that complex neoplastic phenotypes can arise from extremely simple combinations of mutation that, minimally, drive sustained cell proliferation and suppress the concomitant apoptosis.

p53 is the apical effector of a pathway that is inactivated in virtually all human cancer. However, it remains unclear whether the tumor suppressive action of p53 is mediated through its immediate capacity to trigger apoptosis and/or growth arrest, either in response to genotoxic damage or growth deregulation activation, or indirectly through maintenance genome integrity and suppression of emergent neoplastic mutation. To explore these various roles of p53 in tumor suppression and regression *in vivo* we have constructed a mouse in which the endogenous *p53* gene has been replaced with one encoding an ectopically and reversibly switchable p53 protein. Such animals can be rapidly and reversibly toggled between a *p53* null and *p53* wild type state by administration. This model is providing unique insights into the roles of p53 in mediating the DNA damage response, suppressing tumorigenesis and maintaining genome integrity in differing tissues *in vivo*.

© 2004 Cancer Research UK

### S10

### FIBULINS: PHYSIOLOGICAL AND DISEASE PERSPECTIVES

Willam M. Gallagher, Department of Pharmacology, Conway Institute of Biomolecular and Biomedical Research, University College Dublin, Belfield, Dublin 4, Ireland. e-mail: william.gallagher@ucd.ie

The fibulins are an emerging family of secreted glycoproteins characterised by repeated epidermal growth factor-like domains and a unique C-terminal structure (**1**). Members of the fibulin family have been associated with a variety of physiological and disease processes. Fibulins are thought to serve structural roles within the extracellular matrix and have also been shown to modulate cell morphology, growth, adhesion and motility to varying extents. Our research to date has focused primarily on the role of fibulin-1 and –4 in cancer.

Fibulin-4 was originally identified by our group as a candidate oncogene that displays both mutant p53-dependent and -independent oncogenic properties (**2**). In more detail, ectopic overexpression of fibulin-4 increased neoplastic transformation of rat embryonic fibroblasts and promoted tumour cell proliferation *in vitro*. The human fibulin-4 gene was subsequently localised to 11q13 (**3**), a region commonly amplified in a variety of human cancers. In addition, fibulin-4 was shown to be up-regulated at the RNA level in normal versus colon tumour biopsies. Preliminary data has suggested that fibulin-4 may also regulate angiogenesis. However, the functional role of fibulin-4 in tumours *in vivo* has yet to be determined.

We have shown that fibulin-1, the originator of the family, is increased at the protein level in human breast tumours in comparison to normal breast tissue (**4**). Moreover, fibulin-1 was aberrantly processed via differential proteolysis in breast cancer tissues. Our studies have indicated that fibulin-1 may be a useful marker in breast cancer. Four alternative splice variants of fibulin-1 (-1A to –1D) are known, with the –1C and –1D forms being predominantly found. There is now increasing evidence to support the hypothesis that fibulin-1 is a dual oncogene/tumour suppressor gene, with either role dependent on the level and ratio of fibulin-1C and –1D splice variants present (**5**). Using both transcriptomic and proteomic techniques, we are now attempting to elucidate the molecular basis of the differential effect of fibulin-1 alternative splice variants on tumour-associated activities.

Funding for this work is acknowledged from Enterprise Ireland, the Health Research Board of Ireland, the Association for International Cancer Research and the European Commission.

**References**

**1.** Argraves *et al.*, EMBO Reports, *4*: 1127-1131, 2003. **2.** Gallagher *et al.* Oncogene, *18*: 3608-3616, 1999; **3.** Gallagher *et al.* FEBS Letters, *489 (1)*: 59-66, 2001; **4.** Greene *et al.*, British Journal of Cancer, *88 (6)*: 871-878, 2003; **5.** Twal *et al.*, FASEB Journal, *17 (5)*: A1172 Part 2 Suppl. S, 2003.

### S11

### A NEW MOLECULAR TARGET IN COLORECTAL CANCER SIGNALLING: THE *LKB1* TUMOUR SUPPRESSOR GENE AND WNT SIGNALLING

James Spicer[1], Sydonia Rayter[2] and Alan Ashworth[2]
[1] Department of Medical Oncology, Guy's Hospital, St Thomas's Street, London SE1 9RT; [2] Breakthrough Breast Cancer Research Centre, Institute of Cancer Research, Fulham Road, London SW3 6JB

The study of inherited syndromes of cancer susceptibility has proved a valuable route to the identification of many genes involved in human tumourigenesis. Loss-of-function mutations affecting the tumour suppressor gene *LKB1* cause the Peutz-Jeghers syndrome that is associated with a greatly increased risk of cancer. *LKB1* is also inactivated in some sporadic tumours, and encodes a serine/threonine kinase of poorly understood function. Here a new function for LKB1 is suggested. PAR1 is identified as a protein that is phosphorylated downstream of the LKB1 kinase. In turn, via its interaction with PAR1, LKB1 is shown to negatively regulate Wnt signalling. The Wnt pathway, in which a complex of proteins including APC regulates access of the transcription factor beta-catenin to the nucleus, is upregulated in most colorectal tumours. It is proposed that inactivation of LKB1 in PJS releases Wnt signalling from negative regulation, driving formation of colorectal and other tumours. A signalling pathway is thus identified that provides a rationale for the tumour suppressor activity of LKB1, and that suggests a novel therapeutic target.

### S12

### VASCULAR TARGETING STRATEGIES ENHANCE RADIATION THERAPY

Dietmar W. Siemann, University of Florida

Given its pivotal role in tumor development, growth and spread, considerable efforts have been spent on developing therapeutic strategies that compromise the growth and/or function of the tumor neovasculature. Two primary *Vascular Targeting Approaches* are being pursued. *Angiogenic Inhibitors* seek to interrupt the process of angiogenesis to prevent new tumor blood vessel formation. *Vascular Disrupting Agents* aim to cause direct damage to the established vessel network of actively growing tumors. Although vascular targeting approaches have shown significant antitumor efficacy, when used alone, they are unlikely to be curative. Indeed, their greatest utility may ultimately reside in their combination with conventional anticancer treatments. In particular, the application of angiosuppressive or vascular disrupting agents might overcome factors known to adversely affect the efficacy of radiation therapy. These include the metabolic microenvironments associated with the aberrant vascular morphology of solid tumors as well as tumor progression and metastatic spread, two processes dependent on new blood vessel formation. Conversely radiotherapy may serve to eliminate neoplastic cell populations surviving vessel targeting treatment strategies. This presentation will examine the interaction between vascular targeting therapies and radiotherapy and explore issues such as timing, sequencing and the potential of achieving a therapeutic benefit. Combined modality therapies are a mainstay of cancer management and the combination of therapies which target the tumor vessel network in conjunction with radiation therapy holds the promise of providing significant improvements in treatment outcomes and hence should be thoroughly explored.

### S13

### HOW TO COMBINE MOLECULAR THERAPEUTICS WITH RADIATION: LESSONS FROM PRE-CLINICAL ANIMAL MODELS

Kaye J. Williams, School of Pharmacy and Pharmaceutical Sciences, University of Manchester, Oxford Road, Manchester, M13 9PL, UK

A great deal of interest surrounds the development of molecular targeted agents as anti-cancer therapeutics. These have been generated to inhibit specific components of the pathways that promote the survival and proliferation of malignant cells. Although some agents may have utility as stand alone therapies, it is likely that their full clinical potential will be revealed when they are combined with conventional therapy and in particular with radiation. The pre-clinical evaluation of such combinations requires careful consideration, as the influence of each agent on the underlying factors that govern the success of radiotherapy has to be addressed. We have evaluated the use of three molecular targeted agents in a radiotherapy context. ZD1839 (Iressa™; AstraZeneca) and ZD6474 inhibit the tyrosine kinase activities of the epidermal growth factor receptor and vascular endothelial growth factor receptor 2 (KDR) respectively. AG14361 is a novel and potent inhibitor of the DNA-repair enzyme poly(ADP ribose)polymerase. Despite their diverse molecular targets all three agents successfully enhance the response of tumour xenografts to radiotherapy. Rational scheduling designs based upon the potential impact of each agent on the determinants of radiation response (repopulation, reassortment, reoxygenation, repair and radiosensitivity) can enhance the efficacy of the combined therapeutic strategies. In addition to targeting molecular components of the tumour phenotype, tumour specific environmental conditions can also be exploited. Specifically targeting radioresistant hypoxic cells within xenograft tumours affords marked enhancement of radiotherapeutic outcome. These data have clear implications for the future clinical development of combined schedule approaches.

© 2004 Cancer Research UK

### S14

**COMBINING PLATINUM DRUGS AND RADIATION: MECHANISMS AND OUTCOME PREDICTION**

ADRIAN C. BEGG
Division of Experimental Therapy, The Netherlands Cancer Institute, Plesmanlaan 121, 1066CX Amsterdam

The combination of platinum-containing drugs with radiation therapy has become standard treatment for several disease sites and stages. We know, however, that not all patients will benefit from this combination. More information on mechanisms of interaction is needed to choose rationally between current treatment options and to design more effective schedules. This talk will summarize present knowledge on molecular and tissue mechanisms of platinum-radiation interactions, and data on predicting outcome of combination treatments. This will include which DNA repair pathways are involved, whether lesion bypass plays a role, the importance of adduct number and type, the role of GSH, and the effect of treatment-induced changes in tumor subpopulations. Data showing the predictive power of cisplatin-DNA adducts in clinical trials will be shown. Finally I will address questions such as: is synergism important? Why should the combination be tumor specific? Is the use of platinum drugs in such combination therapies ever likely to as effective as molecularly targeted drugs? Is prediction of outcome likely to benefit the patient population as a whole?

### S17

**EARLY LUNG TUMOUR DEVELOPMENT: LONGITUDINAL STUDIES IN PATIENTS, MODELS IN MICE**

Pamela Rabbitts, Department of Oncology, University of Cambridge

In collaboration with Dr Jeremy George, Middlesex Hospital, London we are following a cohort of patients at very high risk of lung cancer development but without detectable tumours at presentation. We use serial fluorescence bronchoscopy to aid the detection of pre-invasive bronchial lesions which provides us with temporally-related high grade lesions some of which eventually become invasive tumours. Biopsies of these lesions are stored both fixed and frozen for later extraction of RNA and DNA following laser capture micro-dissection. We have analysed samples collected in this way to compare molecular genetic changes in pre- and post- invasive lesions using loss of heterozygosity analysis, mutation analysis, comparative genome hybridisation and micro-array expression analysis.

One of the earliest molecular genetic changes identified in pre-invasive bronchial lesions is loss of genetic material from chromosome 3. Using a positional cloning strategy, we have identified a candidate tumour suppressor gene, *DUTT1/ROBO1* from within a region of homozygous loss at 3p12, the smallest deletion being intragenic. To evaluate its role in tumorigenesis, we have precisely recapitulated this deletion in the mouse genome. Mice homozygous for this deletion die at birth from respiratory failure or later with accompanying bronchial hyperplasia. Mice heterozygous for the deletion develop lymphomas and lung adenocarcinomas. The tumours express virtually no Dutt1/Robo1 protein due to methylation of the gene's promoter. We conclude that this gene is acting as a tumour suppressor gene in the development of these mouse lung tumours. Detailed characterisation of the time course of development of these mouse tumours should provide insights in to the very early stages of human lung cancer.

### S18

**VIDEO-ASSISTED THORACIC SURGERY**

David J. Sugarbaker, MD, Brigham and Women's Hospital, Harvard Medical School, Boston, MA 02115.

Minimally invasive techniques have revolutionized the surgical approach to the treatment of lung neoplasms. Video-assisted thoracic surgery (VATS) techniques have proved indispensable for establishing diagnosis of indeterminate pulmonary nodules, and determining stage of lung cancer. In high-risk patients with T1 tumors, VATS wedge resection has been shown to be a safe and sometimes a definitive operation. We recently reported a comparable 5-year survival after wedge resection and anatomic resection in a series of 40 patients with tumors less than or equal to 1 cm. Despite its versatility, the utility of VATS in anatomic lung resections remains controversial. This is further complicated by the fact that the techniques reported in the literature are not uniform. Four different approaches to VATS lobectomy have been described. Morbidity and mortality in several published series, however, compare favorably to open surgery. The 3-year survival in published series for stage I disease ranges from 80% to 94%. The popularity of VATS is both surgeon and patient driven and can be attributed to several factors: improved postoperative recovery, decreased length of stay, less pain, improved pulmonary function, and cosmetically better results. Despite these advantages, there is a learning curve associated with VATS resections. The VATS approach is contraindicated for centrally located tumors and bulky mediastinal disease. However, for a select cohort with stage I or early stage II disease without prior therapy, we advocate VATS lobectomy as the standard of care.

### S19

**RNA POLYMERASE III TRANSCRIPTION – A BATTLEGROUND FOR ONCOGENES AND TUMOUR SUPPRESSORS**

Robert J. White
Institute of Biomedical and Life Sciences, Davidson Building, University of Glasgow, Glasgow, G12 8QQ, U.K.

Transcription by RNA polymerase III (pol III) is abnormally active in transformed and tumour cells. Our work aims to characterize the mechanisms responsible for this deregulation.

The tumour suppressor RB inhibits pol III transcription by binding and inactivating TFIIIB, a factor which recruits pol III onto promoters. RB function is compromised in cancers through one of three mechanisms: mutation of the *Rb* gene; hyperphosphorylation by cyclin-dependent kinases; or binding of oncoproteins. Each of these mechanisms can derepress TFIIIB and increase pol III activity. TFIIIB is also bound and repressed by p53 in healthy cells. Mutations in p53 contribute to the deregulation of pol III transcription in some cancers and in Li-Fraumeni syndrome. Mdm2 and E6 activate pol III transcription by neutralising p53.

As well as being regulated by these tumour suppressors, TFIIIB is also bound and activated by several oncogenic proteins, including c-Myc, CK2 and the Erk MAP kinases. TFIIIB therefore lies at the centre of a complex regulatory network of conflicting influences. The fact that it is targeted directly by oncoproteins and key tumour suppressors provides a clear indication of the importance of controlling pol III transcriptional output.

In support of this, pol III-specific transcription factors are frequently overexpressed in human tumours. For example, the DNA-binding factor TFIIIC2, which recruits TFIIIB to promoters, is produced at high levels in ovarian carcinomas. HPV16-infected cervical carcinomas overexpress Brf1, a specific subunit of TFIIIB. Remarkably, Brf1 overexpression can be sufficient to accelerate cell growth and proliferation. Clearly, there is strong selective pressure to raise pol III output during tumour development; this can be achieved through a range of molecular mechanisms and may have dramatic effects on proliferative capacity.

© 2004 Cancer Research UK

### S20

**NF-KB: TUMOUR PROMOTER OR TUMOUR SUPPRESSOR?**

Neil D. Perkins, Division of Gene Regulation and Expression, School of Life Sciences, University of Dundee, MSI/WTN Complex, Dow Street, Dundee DD1 5EH

Although typically associated with activation by inflammatory cytokines, NF-κB subunits are also activated by many common cancer treatments and can be found aberrantly active in human tumours. We have investigated the relationship between the NF-κB transcription factor family and the ARF/p53 tumour suppressor pathway. In addition, we have investigated p53-independent mechanisms regulating NF-κB function following stimulation with cytotoxic stimuli, including chemotherapeutic compounds. We find that NF-κB subunits can function in two ways. In one sense, NF-κB subunits behave in an oncogenic manner, inducing the expression of anti-apoptotic and pro-proliferative genes. In other circumstances, however, the opposite occurs and NF-κB subunits actively repress the expression of these same target genes. We have discovered that p53, ARF and some chemotherapeutic drugs have the ability to "switch" NF-κB subunits from being oncogenic to this more tumour suppressor-like function. Taken together, these results indicate that in the early stages of oncogenic transformation, NF-κB could act to inhibit tumour growth. As tumours develop, however, and tumour suppressor genes become inactivated, NF-κB will revert to its oncogenic function. We suggest that care should be taken when designing anti-cancer treatments based on inhibition of NF-κB since under some circumstances, NF-κB might inhibit rather than stimulate tumour growth.

### S21

**TRANSCRIPTIONAL REGULATION BY THE WILMS' TUMOUR SUPPRESSOR PROTEIN WT1.**

Kelly Addison, Katherine Boylan, Jörg Hartkamp, Laura Green, Kate Wagner and Stefan Roberts.
Gene Expression and Cell Signalling Laboratories, School of Biological Sciences, University of Manchester, Manchester M13 9PT.

Wilms' tumour is a paediatric malignancy of the kidneys that affects 1 in 10,000 children. The Wilms' tumour suppressor protein WT1 is mutated in approximately 15% of Wilms' tumours, while others show aberrant WT1 expression. The WT1 protein contains four zinc fingers at its C-terminus and an N-terminal region that harbours the transcriptional regulatory domains. The WT1 message is alternatively spliced, which results in the generation of several isoforms that can have different physiological effects.WT1 can either activate or repress transcription and this is dependent upon the isoform of WT1 and the specific cellular context. Our work concerns the analysis of transcriptional regulation by WT1 and the interaction partners that modulate WT1 function. We have found that the apoptosis regulator Par-4 can act as a coactivator for a specific isoform of WT1 and that this function is required for the ability of WT1 to mediate cell survival following pro-apoptotic insult. Par-4 can cooperate with WT1 to stimulate transcription of the Bcl2 promoter, providing a mechanistic basis for this effect. Our current studies suggest that WT1 isoform imbalance and aberrant Par-4 expression might be involved in the formation of some Wilms' tumours. We have also isolated a transcriptional cosuppressor of WT1, BASP1. BASP1 silences WT1-mediated transcriptional activation. Consistent with this function, down-regulation of BASP1 by siRNA results in activation of the amphiregulin promoter by WT1. Amphiregulin is a potent inducer of kidney tubule branching, suggesting that BASP1 might modulate the function of WT1 in kidney development. Indeed, BASP1 shows both temporal and spatial co-expression with WT1 in the embryonic kidney.

### S22

**TARGETING HYPOXIA INDUCIBLE FACTOR-1.**

Noan-Minh Chau, Paul Rogers, Wynne Aherne, Ted MacDonald, Paul Workman, and Margaret Ashcroft.
Cancer Research UK Centre for Cancer Therapeutics, The Institute of Cancer Research, Surrey, United Kingdom.

Hypoxic regions are often found within solid tumours. One of the proteins that is induced in response to low oxygen levels is hypoxia inducible factor-1α (HIF-1α). In response to hypoxic stress HIF-1α dimerises with HIF-1β, a constitutively expressed protein, to form the heterodimeric transcription factor, HIF-1. HIF-1 binds to a short DNA sequence within the promoter or enhancer region of specific target genes. This is called the hypoxia response element (HRE). HIF-1 target genes include VEGF, glycolytic enzymes, glucose-transporters and erythropoietin; these are important in tumour progression and metastasis. HIF-1α has been shown to be overexpressed in a number of cancers, which is associated with agressive tumours and treatment resistance. HIF-1 is an attractive target for therapeutic intervention.

We have generated U2OS human osteosarcoma cells stably expressing a luciferase reporter construct under the control of a consensus HRE (pGL-HRE). We have used this cell-based system to perform high-throughput screening to identify compounds that inhibit HIF-1 activity induced by treatment with the hypoxia mimetic, DFX (500mM) for 16 hours. Initially, we performed a pilot screen of 4500 compounds, which included the NCI 'Diversity Set' of 2000 compounds. 'Hit' compounds from this screen have been evaluated in a variety of secondary assays. These studies have indicated that molecular targeting of the HIF-1 pathway may be achieved in multiple ways. A large-scale screen is complete and we are currently evaluating 'hit' compounds. This system has proved to be a useful tool to identify HIF-1 inhibitors and evaluate compounds that may be therapeutic candidates for drug development.

© 2004 Cancer Research UK

